# Capacitive Neuromodulation via Material-Based Passive Interaction: Efficacy in Motor Function Improvement in Parkinson Disease

**DOI:** 10.3390/bios14070354

**Published:** 2024-07-20

**Authors:** Fabrizio D’Errico, Francesco Serio, Gianluigi Carioni

**Affiliations:** 1 Politecnico di Milano, Department of Mechanical Engineering, Via La Masa 1, 20156 Milan, Italy; 2 ASL Taranto, Viale Virgilio, 31, 74121 Taranto, Italy; 3 A.P.R Meds Srl, Via Ugo La Malfa 3, 26900 Lodi, Italy

**Keywords:** neuromodulation, Parkinson’s disease, dielectric interaction, neurotherapy

## Abstract

A non-invasive and non-pharmacological approach is evaluated for the proprioceptive and postural improvement of PD subjects. The authors evaluated the effectiveness of a class I medical device according to EU regulation 745/2017 designed to develop the mechanism of action based on the modulation of action potentials, which occurs in prevalent pathways of the afferent peripheral nervous system efferent in subjects with spasticity. The present observational study, structured in a double-blind randomized manner, therefore, had the main aim of evaluating the ability of the device to improve on the motor and proprioceptive function of PD patients. This study was based on the instrumented gait analysis performed according to the Timed Up and Go (TUG) test procedure, as well as using a fall risk assessment in accordance with the Berg Balance Scale (BBS) procedures. This study involved 25 participants in the active group (no placebo) and 25 in the non-active group (placebo), the latter to whom non-functional devices were applied, but in every respect identical to the functional devices applied to the 25 patients in the no placebo group. Data analysis was conducted using statistical methodologies for statistics, the statistical significance of the results for the observed samples and the interdependence between the measured variables. The study of the mechanism of action based on the remodulation of action potentials was preliminary conducted through numerical modeling of the Hodgkin–Huxley axon, modified by introducing the influence of the capacitive device applied in clinical tests into the validated model to target the dielectric properties of materials constituting the passive sensor. The use of the neuromodulation device promises observable improvements in motor function among PD patients, including increased limb mobility and greater postural stability.

## 1. Introduction

In proprioception, the good functioning of the feedback circuit created by the electrochemical signals propagating along the neuronal fibers is a key and crucial element for the correct management of the spatial movement of the limbs at both a conscious and unconscious level [1,2]. At the same time, abnormal ion concentration gradients, mainly Na^+^ and K^+^, are a determining factor in the production of motor perturbation in conditions of neurodegenerative pathologies such as Parkinson’s disease. As well, the disordered ion concentration gradients promoted by active ion transport, mainly to Na^+^ and K^+^, are critical for exploring therapeutic interventions for neurodegenerative conditions such as Parkinson’s disease [2]. Through the activity of voltage-gated ion channels, changes in membrane potential generate action potentials in the form of “trains” of waves that travel along afferent pathways to the central nervous system that modulate the activity of motor neurons, regulate contraction muscle and maintain postural stability through adequate proprioceptive mechanisms [3,4]. The modulation of action potentials, influenced by factors such as the dynamics of ion channels and the activity of neurotransmitters, is particularly relevant in the management of muscle tone, and lends itself to insights into the complex interactions between neurophysiological mechanisms and perceptual experiences [5].

Techniques such as deep brain stimulation (DBS), although very invasive, which involves high-frequency stimulation of specific brain regions, have proven promising in the modulation of neuronal activity in Parkinson’s subjects [6]. Non-invasive approaches, such as local mechanical vibration (FMV), are based on postural and proprioceptive improvement using local vibrational stimuli capable of strengthening proprioceptive feedback [7,8]. Likewise, non-invasive techniques such as thermal blocking of action potentials using infrared sources, direct modulation of the membrane potential using an external electric field and indirect modulation of the membrane potential using an external variable magnetic field have been studied. In particular, with regard to the thermal block of action potentials, the study by Mohit Ganguly et al. [9] based on Hodgkin–Huxley modelling with the introduction of the localized thermal effect into the model, demonstrated that the increase in temperature accelerates the activation of voltage-gated potassium ion channels, leading to a hyperpolarization condition, causing the action potential block.

An external electric field can modify the membrane potential, which is typically mediated by the extracellular electrolyte fluid, thus influencing the opening or closing of voltage-gated channels. Ions into and out of the cell can be moved directly by the electric field, which influences the concentration of the ions and subsequently alters the dynamics of the channel, as observed in the work of Mortimer and Bhadra on monopolar anodal stimulation [10]. Speculations about time-dependent magnetic fields have been exploited in practical non-invasive applications of ion cyclotron resonance (ICR), a method that permeates variable magnetic fields within tissues. According to the principles outlined by Abraham R. Liboff [11], Faraday’s law of induction suggests that a changing magnetic field can induce an electromotive force (EMF) within the electrolyte surrounding a neuron. Liboff’s insights into the interaction of cyclotron resonance with membrane-bound ion channels suggest that this mechanism could influence gating processes that control ion flux.

The literature on the efficacy of non-invasive approaches in the management of Parkinson’s disease (PD) has therefore ranged from local vibrational stimulation and modulation of membrane potential by external electric and magnetic fields [8,9,10,11]. These approaches have demonstrated significant improvements in motor functions and postural stability, providing a solid basis for further research and development of non-invasive, autonomous and wearable devices, whose mechanism of action is focused—at a hierarchical level—on the most proximal causes of uncontrolled or poorly modulated neurostimulations, as occurs in Parkinson’s patients.

Therefore, this study introduces the use of the Equilibrion^®^ device, a novel non-invasive passive sensor that detects and responds to an input from the physical environment, specifically the concentration of ionic charges in the extracellular fluid of the peripheral neuronal membrane, and responds with an action mechanism based on the dielectric properties of the material preliminary researched with numerical approaches. The Equilibrion^®^ class I medical device, compliant with Regulation (EU) 2017/745, through a capacitive mechanism detailed in this study that has recently received the title of industrial invention patent in Italy and is currently in consideration for EU and USA patent office (still in the pending phase) provides local interaction with the skin to modulate the ionic environment around neuronal membranes. The evaluation has been conducted through a double-blind, double-dummy, randomized controlled observational study (RCOS). This study aimed at systematically collecting clinical sample data on the CE marked device, therefore employing medical device according to indications in a context of normal clinical practice, namely excluding interventions outside of what is claimed in instructions to patients. By measuring and observing improvements in limb mobility and postural stability, we aimed to statistically evaluate the effectiveness of intervention of a non-pharmacological supportive therapy for PD patients. Specifically, this study investigated the enhancement of limb mobility and postural stability in patients with Parkinson’s disease, namely the potential impact on quality of life on individual clinical cases.

## 2. Materials and Methods

Designed for skin application, the device aims to rebalance ionic charges within the fluid environment of neuronal membranes. Fabricated by A.P.R. Meds srl (Italy), it is a registered Class 1 medical device categorized for neurological physiotherapy, in accordance with European Directive 93/42/EEC–2007/47/EEC, registered with the Ministry of Health under registration number 2434444, protected by industrial patent in Italy, invention patent no. 102022000011933, currently in pending state for extension in USA and EU.

**Figure 1:**
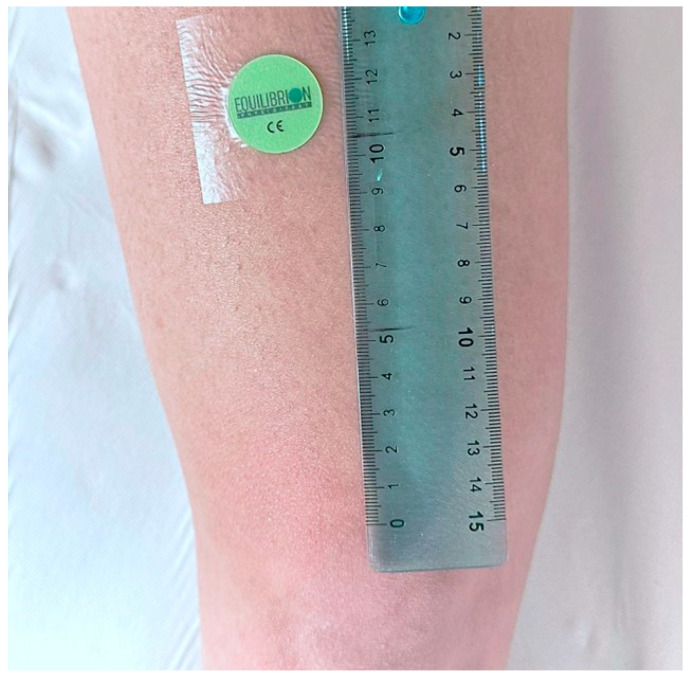
Example of a skin device application to the bilateral rectus femoris muscle belly on the left leg.

It consists of a 25 mm circular plate (Figure 1), composed of layers of inert and biocompatible polymeric materials with a specific dielectric constant within the range of relative dielectric constant at 1 MHz, from 2.0 to 5.0.

The mechanism of action of the medical device is strictly correlated to the dielectric properties obtained by assembling 12 layers of specific polymers to achieve the target dielectric constant, based on preliminary studies conducted using the axon numerical model, as depicted below.

A precise dielectric feature is fundamental for the proper interaction with the peripheral nervous system at those target points therapist identify for PD patients based on standardized sensory area targets, as discussed in detail in the Section 4 .

Regarding the capacitive device’s mechanism of action, it was important for the therapists involved in this study to better understand the key foundations of the device. These foundations were the objectives of the research and development results at the bioengineering and material specialists’ perspectives, just summarized in this work. Therefore, the present work present dual methodological approach:

(a) Development of numerical modelling of an axon using the Hodgkin–Huxley differential equation model 12 that allowed developers in selecting property of materials constituting the medical device for the desired interactions with neuronal membranes.
(b) Observational study to point out effectiveness level on significative samples with Parkinson Disease patients selected under constraints of study protocol, in the following discuss in depth.

### 2.1. The Numerical Model

**Figure 2:**
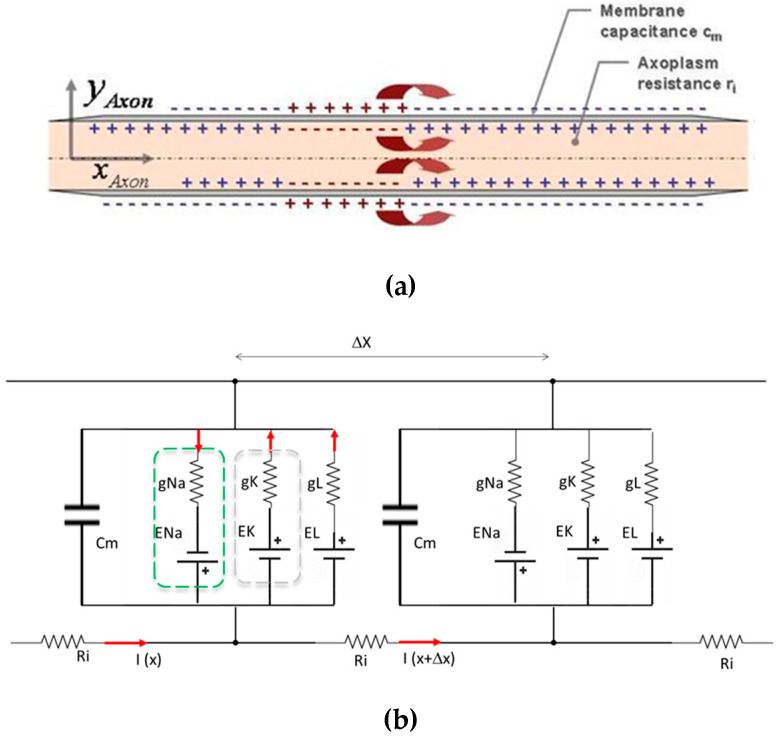
Schematic of axon modelling: (**a**) diagrammatic representation of an axon; (**b**) serial segments of equivalent electric circuits of Hodgkin–Huxley.

During the development phase, the device was preliminarily studied and numerically validated by developing a numerical model in a MATLAB environment of the baseline Hodgkin–Huxley equivalent circuit (also HH, in the following) (see Figure 1), subsequently modified with the appropriate insertion of a capacitor or a generator of current, which models the presence of an external element with dielectric behavior (see Figure 2).

The Hodgkin–Huxley model [12] is analogous to a simple electrical circuit with batteries, resistors, and capacitors. It describes the flow of ionic currents through the neuron’s membrane, which can be modelled as resistors, and the capacitive properties of the membrane. The model focuses on the movement of sodium (Na^+^), potassium (K^+^), and leak currents through specific ion channels. When sodium channels open, Na^+^ flows into the neuron, causing depolarization and generating an action potential. Subsequently, sodium channels close and potassium channels open, allowing K^+^ to exit the cell, leading to repolarization and then hyperpolarization, returning the membrane to its resting potential. The membrane’s conductance is determined by the probability that the ion channels are open, which is voltage dependent. Hodgkin and Huxley developed differential equations to describe these probabilities and their dynamics over time. The conductance of ion channels, such as sodium and potassium, increases in response to specific voltage changes.

In preliminary studies for defining the design of the device capable to interact with electric (in reality, they are electrochemical signals in the real axons) signals with the Hodgkin–Huxley model, a numerical model implemented in MATLAB was constructed to simulate action potential propagation along an axon. Key steps included:

1. *Parameter initialization:*
  - Cm = 0.5; μF/cm^2^, original membrane capacitance;
  - gNa = 120; mS/cm^2^, maximum conductance of the sodium channel;
  - gK = 36; mS/cm^2^, maximum conductance of the potassium channel;
  - gL = 0.3; mS/cm^2^, leak conductance;
  - ENa = 50 mV, sodium equilibrium potential;
  - EK = −77; mV, potassium equilibrium potential;
  - EL = −54.387; mV, leak equilibrium potential.
2. *Simulation:*
  - The axon was divided into segments, and the differential equations were solved using numerical integration with a time step (dt = 0.01 ms).
  - An additional capacitor (C_user ) was introduced in parallel to the membrane to observe its effects on action potential propagation.
3. *Action Potential Generation:*
  - The model describes how the opening of sodium and potassium channels leads to the generation and propagation of action potentials.
  - The probability of ion channel gates being open is dependent on the membrane voltage, and these probabilities were calculated using the Hodgkin–Huxley equations. Main equations implemented in Matlab code were:

*Membrane potential equations:*

(1) $$C_{tot}\frac{dV}{dt}=I-I_{Na}-I_{K}-I_{L},\;\mathrm{with}\;C_{tot}=C_{m}+C_{user}$$

*Ionic current equations:*

(2) $$I_{Na}=gNa\cdot m^{3}h\cdot (V-E_{Na}),$$

(3) $$I_{K}=gK\cdot n^{4}\cdot (V-E_{K}),$$

(4) $$I_{L}=gL\cdot (V-E_{L}),$$

*Gating variables, for ion channels opening regulation:*

(5) $$\frac{dm}{dt}=\alpha_{m}\cdot (1-m)-\beta_{m}\cdot m$$

(6) $$\frac{dh}{dt}=\alpha_{h}\cdot (1-h)-\beta_{h}\cdot h$$

(7) $$\frac{dn}{dt}=\alpha_{n}\cdot (1-n)-\beta_{n}\cdot n$$

and coefficient *α* and *β* depending on membrane potential V and fixed parameters. All parameters have been selected considering healthy humans, specifically the maximum conductances of the ion channels (gNa, gK, gL), and the equilibrium potentials (E_Na_, E_K_, E_L_). These values are based on well-established physiological data from classic neuroscience research [12,13,14,15,16,17].

**Figure 3:**
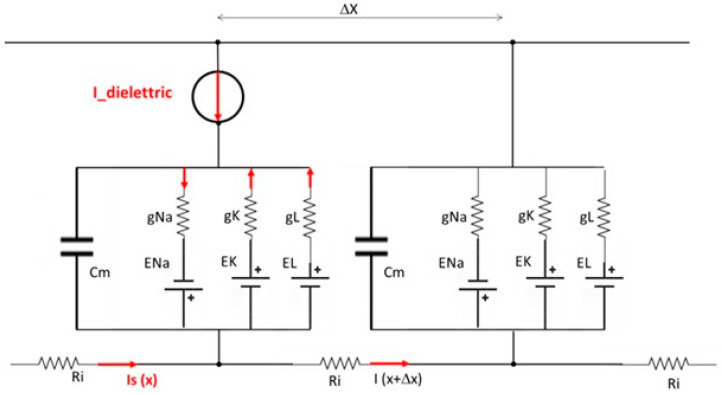
Diagram of the series of Hodgkin–Huxley electric equivalent circuits of the modified model with the intermediate presence of a dielectric element, modelled with an I-dielectric impressed current generator in opposition to the stimulus current Is.

4. *MATLAB Simulation Code*
  - Based on the developed model, experiments were conducted to verify that in the presence of certain parameters of the dielectric, the capacitive element placed on the skin is capable of interacting with the ionic environment that envelops neuromembranes, thus modulating neuronal action potentials within the central nervous system.
  - The axons within this model were divided into discrete segments to allow precise measurement of the threshold block length throughout the axonal model (Figure 2).
  - The model incorporated a simulated microcurrent generator to mimic the effect of an external capacitive and dielectric element (Figure 3), simulated in the Hodgkin–Huxley varied model a current generator), reflecting what produced in dielectric materials due to electrostatic induction realigning polar molecules in response to electric fields.

### 2.2. Methods and Procedures of Observational Study

A double-blind, double-dummy, randomized controlled observational study investigated the effectiveness of a standard balance training program combined with the use of the wearable proprioceptive stabilizer Equilibrion^®^. This study did not involve any interventions beyond the normal clinical practice described in the product leaflet. Patients were recruited in accordance with stringent ethical guidelines; informed consent was obtained from all enrolled patients, ensuring that they were fully informed about this study’s purpose. Personal and health data gathering procedures that were processed in compliance with GDPR (EU Regulation 2016/679) and national legislation, ensuring anonymization and secure storage. Participant documents were kept in a secure location, identified only by a code known to researchers. Anonymized data were available for regulatory review and scientific publications. The study protocol was reviewed and approved by the internal Ethics Committee of manufacturer as the body entity responsible of conformity to EU Regulation 745/17 of the Class I medical device, thus ensuring adherence to Good Clinical Practice and Helsinki Declaration principles. The study followed principles of phase III clinical observational study targeting Parkinson’s patients with specific clinical characteristics to evaluate the therapeutic support provided by the passive sensor device, in combination with standard pharmacological and physiotherapy treatments. Moreover, patients were informed that this study aimed to accurately measure the effects of the new treatment compared to existing treatments and they would be randomly assigned to receive either the experimental treatment or a placebo, which contains no active ingredient. As an auxiliary therapy to improve Parkinson’s disease symptoms, posture, movement stability, and reduce the risk of falls, patients did not modify their own standard pharmacological and physiotherapeutic treatments. Unique requirements and the application of the therapeutic wearable passive sensor occurred when the effects of the previously administered pharmacological treatment began to wear off, specifically before the next scheduled dosage. Patients also were informed no risks have been described in the technical documents and material specifications provided by the manufacturer for class I medical devices, except for a skin allergy due to the patch used to apply the device.

This study enrolled 50 patients who were randomized into one of the following groups:

- 50/2 patients in the experimental Group PD/EQ (Parkinson Disease patients with Equilibrion^®^ therapy).
- 50/2 patients control Group PD/P (Parkinson Disease patients with Placebo).

The two groups underwent the following program.

- The active group PD/EQ: PD patients were treated with an innovative postural stabilizer device (Equilibrion) for 30 min before postural check with Geit Analysis parameters.
- The non-active group PD/P: PD patients treated with a placebo device (similar to Equilibrion but inactive) for 30 min before postural check with Geit Analysis parameters.

Five functional and non-functional devices were applied to the skin of participants in both the active (non-placebo) and non-active (placebo) groups using a medical band, following specific standardized Sensory Area Targets:

Position 1: the 7th cervical vertebra;
Position 2: the bilateral rectus femoris muscle belly on the right leg;
Position 3: the bilateral rectus femoris muscle belly on the left leg;
Position 4: the bilateral gastrocnemius muscle on the right leg;
Position 5: the bilateral gastrocnemius muscle on the left leg.

The application positions follow the protocol developed by the medical therapists at fabricant A.P.R. Meds Srl. This protocol is fundamentally based on identifying areas at the five application points (By most clinical-therapeutic details: 1. C7 (7th cervical vertebra), as it stimulates the head-righting reflex. The cervical region is rich in proprioceptors, which helps to alleviate upper limb adiaconcinesia, improving the kyphotic posture of the upper trunk (camptocormia). 2. Bilateral Rectus Femoris Muscle Belly: Muscle rigidity in patients with extrapyramidal syndrome is a clinically significant symptom that affects the complete extension of the knees, compromising gait and static and dynamic stability. 3. Bilateral Triceps Surae Muscle: Given that plastic rigidity predominates in the muscular system of patients with extrapyramidal syndrome, these points were identified to free the ankle joint, enhancing its flexion–extension, thereby reducing foot dragging, freezing episodes, and the risk of falls). that are functionally and purposefully involved with motor neurons and peripheral nerve bundles. This is because in PD patients, there is a reduction in dopamine production by the putamen. Dopamine is crucial for regulating the transmission of nerve impulses. The decreased dopamine levels result in altered nerve signal transmission, often transmitted as unmodulated bursts of action potentials. In PD patients, these areas are affected by disrupted and spastic signals, which impair posture and mobility. Therefore, the 5 positions are selected as the most critical points for intervention, as the effect of the capacitive wearable device would progressively modulate the action potential trains that are transmitted to key muscles responsible for posture and dynamics in PD patients.

After the application of the wearable skin devices, patients were asked to sit for 30 min to allow for CNS interaction to commence. The T1 motor impairment TUG (Timed Up and Go) test was then initiated for data acquisition.

Inclusion criteria for all patients admitted to the Centro Fisioterapico Medico C.F.M Srl in Treviglio (Italy) were:

- Parkinson’s disease stage II–III, evaluated with the H&Y scale,
- Presence of postural alterations,
- Presence of postural instability
- Ability to participate in physiotherapy,
- Absence of cognitive impairment (MMSE > 24/30), and
- Stable medications.

Exclusion criteria:

- Presence of DBS;
- Severe cardiac and/or pulmonary disease;
- Patients underwent physiotherapy treatment while in an “off medication” state, meaning that the effects of levodopa had worn off.

The study coordinator responsible for statistical analysis was blinded to the group allocation. The therapist providing the interventions was blinded to the allocation of functional and non-functional devices.

Both functional (applied to the active group) and non-functional (applied to non-active group) wearable devices were identical, not distinguished by their external appearance, and the constituent inert materials used for non-functional devices did not target the specific range for the dielectric constant and insulating parameters that allow functional response. Furthermore, as a passive device, neither functional nor non-functional devices caused any recognizable sensory sensation, guaranteeing patients’ blindness.

### 2.3. Clinical Assessment of Admitted Patients

Baseline data included age, gender, body mass index (BMI), disease duration, anti-PD medications expressed as levodopa daily dose (Table 1), cognitive status assessed using the Mini-Mental Status Exam (MMSE) or MMSE [18], and progression of Parkinson’s disease symptoms assessed using the Hoehn and Yahr Scale [19].

Table 1 summarizes the characteristics and clinical data from the active group (*n* = 25) with the placebo group (*n* = 25) of patients with Parkinson’s disease (PD). Clinical parameters are: body mass index (BMI), disease duration, Mini-Mental State Examination (MMSE) scores, daily levodopa dose (LED), age, and the Hoehn and Yahr (H&Y) scale. Based on the statistical analysis results:

- The mean BMI of the active group was slightly higher than that of the placebo group, but the Mann–Whitney U test yielded a *p*-value of 0.685, indicating no statistically significant difference between the two groups in terms of BMI.
- Disease duration: Both groups had a similar mean disease duration, with a Mann–Whitney U *p*-value of 0.624, suggesting no significant difference in the duration of PD diagnosis.
- The MMSE measures cognitive impairment, with higher scores indicating better functioning. The mean MMSE score of the active group was marginally higher than that of the placebo group, but the *p*-value of 0.282 from the Mann–Whitney U test suggests no significant cognitive performance difference between the groups.
- The daily levodopa dose (LED) was obtained from the pilot study. The average daily levodopa dose was slightly higher in the active treatment group than that in the placebo group. However, a *p*-value of 0.727 indicated no significant difference in medication dosage between the two groups.
- There was a noticeable difference in the mean age between the active (77.56) and placebo groups (67.15). The Mann–Whitney U test yielded a *p*-value of approximately 0, indicating a statistically significant difference in age between the two groups. This could result in a potentially critical finding in this pilot study, potentially affecting the outcomes and interpretation of treatment effectiveness.
- The Hoehn and Yahr scale was used to describe PD progression. The scores were similar between the groups, with no significant differences observed (chi-square *p*-value of 0.607).

## 3. Results

**Figure 4:**
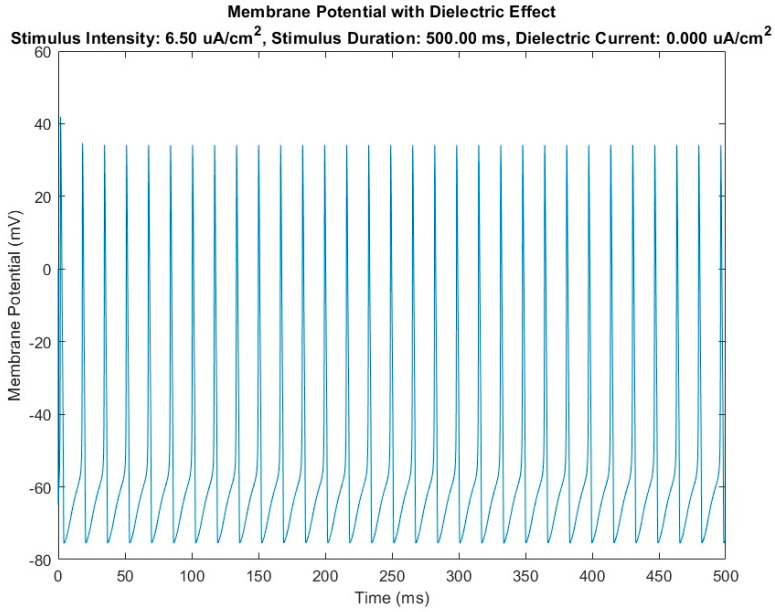
Diagram of membrane potential variation over time in the case of the HH-baseline model (without the introduction of the dielectric element) in the presence of a stimulus current Is.

**Figure 5:**
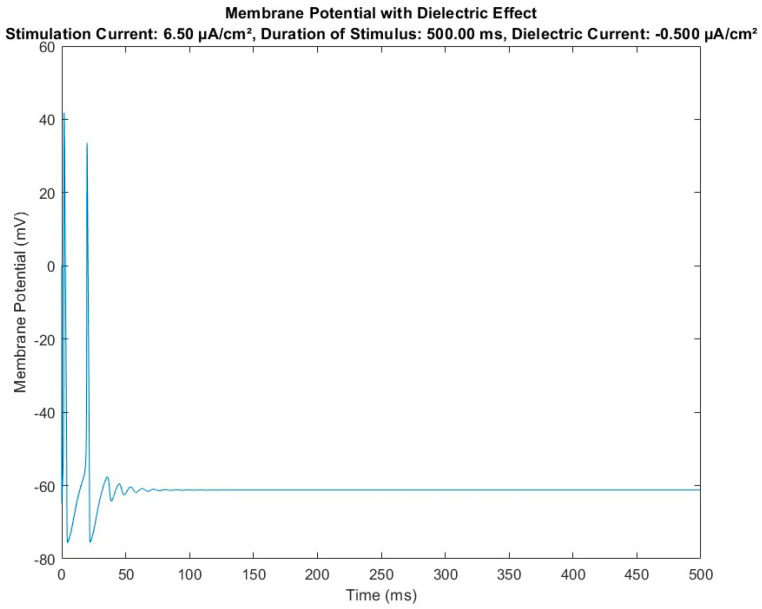
Diagram of membrane potential variation over time in the case of the HH-modified model (with introduction of the dielectric element) in the presence of a stimulus current Is.

The results of numerical model adopted to investigate effect of a capacitive passive sensor device are summarized in Figure 4 and Figure 5.

Figure 4 shows the trend of the membrane potential recorded over time at node 5, which is the median node of the HH-baseline numerical model. It is evident that, in the presence of a certain stimulus current density fixed in the numerical simulation trials at 6.5 μA/cm^2^, the action potential is activated, developing a train of action potentials (see repetitive peaks of potential in figure) that pass over time through node 5.

Figure 5 shows the results obtained with the same constitutive parameters of the HH-baseline model and the same external input parameters (i.e., stimulus current density at 6.5 μA/cm^2^ and same observation time, 500 milliseconds) but using the HH-modified numerical model reported in Figure 3, highlighting the effect on the model of dielectric module insertion. The result was a modulation (in terms of amplitude and frequency) of the action potentials in transit from the node where the dielectric element was inserted.

### 3.1. Experimental Clinical Trials Observations

Motor impairment follows the Timed Up and Go (TUG) [20] procedure for instrumented motor examination. The measurements performed using the G-Walk wearable inertial sensor device were investigated for the most critical motor impairment parameters listed in Table 2.

The Berg Balance Scale (BBS) [21] was employed to evaluate balance and risk of falling in patients with Parkinson’s disease. The Berg Balance Scale (BBS) is a standardized clinical tool used to assess balance in patients with movement disorders. It consists of 14 tasks, each scored on a scale from 0 to 4, with higher scores indicating better balance. Some of the tasks are:

- Sitting to Standing: The patient is asked to stand up from a seated position without using their hands for support, assessing their ability to transition smoothly between sitting and standing.
- Standing with Eyes Closed: The patient stands with their eyes closed for 10 s, testing their balance without visual input.
- Reaching Forward with Outstretched Arm: The patient reaches forward as far as possible without moving their feet, evaluating their ability to maintain stability while extending their arm.
- Retrieving Object from Floor: The patient picks up a small object from the floor while standing, assessing their ability to bend and return to an upright position without losing balance.
- Turning 360 Degrees: The patient turns in a full circle, first to the right and then to the left, measuring their ability to maintain balance while changing direction.

These tasks collectively help in evaluating various aspects of balance, including static and dynamic stability, and provide a comprehensive assessment of the patient’s balance abilities.

Table 3 shows the final outcomes of the paired *t*-test analysis conducted to evaluate the differences in Berg Balance Scale (BBS) scores from T0 (pre-treatment) to T1 (post-treatment) within the active and non-active groups. The paired *t*-test statistical method was used to compare BBS collective results of two samples (active and non-active groups) furthermore comparing the effect of the tasks performed before (T0) and after (T1) the treatment on the same subjects.

### 3.2. Results of the Instrumented TUG Trial Test for the Active Group with Equilibrion

Statistical analysis of the data collected from the TUG tests was conducted on the active group of 25 patients who underwent therapeutic treatment. The measured parameters considered (P1-P11, refer to Table 2) were analyzed with the Shapiro–Wilk method (refer to Table 4) and test *t*-statistic (refer to Table 5) to verify, respectively, normality and differences pre- and post-treatment.

### 3.3. Results of the Instrumented TUG Trial Tests for the Non-Active Group (Placebo Group)

Similarly, statistical analysis of the data collected from the TUG tests was conducted on the non-active group of 25 patients who underwent placebo treatment; the measured P1–P11 parameters (refer again to Table 2) were analyzed with the Shapiro–Wilk method (refer to Table 6) and test *t*-statistic (refer to Table 7) to verify, respectively, normality and differences pre- and post-treatment.

## 4. Discussion

The non-pharmacological approach employed in the pilot study involved a medical device based on polymeric material multilayers with a specific dielectric constant to passively modulate the repolarization of the neuronal membrane, thus representing a non-invasive therapeutic strategy for a spectrum of neurological conditions. Dielectric materials are substances that do not conduct electricity due to the random orientation of their constituent molecules. However, they can become polarized in response to the presence of electric charges or when immersed in an ionically charged electrolyte, creating internal electric dipoles. Because of their ability to orient these dipoles, dielectric materials are employed in capacitors to store electrical energy and also act as electrical insulators. In fact, inserting a dielectric between the conductors of a capacitor increases its capacitance. This occurs because the polarization of the dielectric reduces the electric field between the conductors, allowing for a greater amount of charge to be stored for the same applied voltage.

Among the characteristics of materials, the dielectric constant is the most relevant parameter that measures a material’s ability to store electrical energy. It indicates the capability of internal dipole orientation under external electric fields. Higher values indicate a greater capacity for energy storage.

When a dielectric material is immersed in a positively charged electrolyte solution, an electric double layer (EDL) [22,23] forms at the interface between the dielectric and the solution. This process involves the creation of two distinct layers of opposite charges [18,19]:

- *The Helmholtz Layer (Inner Layer)*: This is the first layer that forms directly on the surface of the unpolarized dielectric. Positive ions in the electrolyte move towards the dielectric’s surface. In response, negative charges within the dielectric align towards the surface to balance the positive charges. This results in a zone of positive charges in the electrolyte and a zone of negative charges on the dielectric’s surface, forming the initial part of the electric double layer.
- *Diffusion Layer (Outer Layer):* This second layer extends into the electrolyte solution beyond the Helmholtz layer. Here, the positive ions are more weakly bound to the dielectric surface and gradually spread out into the solution. In this layer, the concentration of positive ions decreases with distance from the dielectric surface, forming a gradual distribution of charges.

The establishment of the electric double layer has direct effects on both the dielectric and the electrolyte:

- *Dielectric:* The dielectric molecules polarize, with negative charges aligning towards the surface to balance the positive ions in the electrolyte.
- *Electrolyte*: Positive ions accumulate near the dielectric surface, attracted by the negative charges on the dielectric.

This intrinsic property of dielectric materials and their interactions with electric or electrochemical states is crucial for analyzing the behavior of specific dielectric materials in various applications, including their role in therapeutic medical devices.

Based on above premises, the mechanism of action of the medical device used in this observational study is primarily based on its own dielectric constant, investigated originally with numerical approach, which enables the capacitive device applied to the skin to interact with membrane extracellular electrolytes by exploiting ion charges.

**Figure 6:**
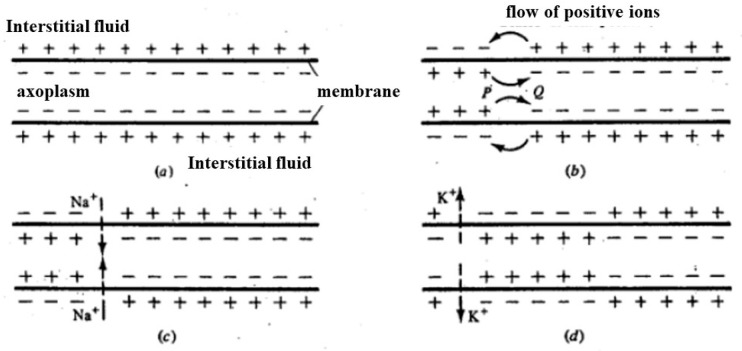
(**a**) Based on the resting potential, positive charges accumulate on the outer part of the axon membrane; (**b**) once the depolarization of the membrane is locally triggered (i.e., is above a threshold), the distribution of charges locally inverts; (**c**) in correspondence with adjacent areas where membrane depolarization has occurred, the potential increases, passing above the threshold, causing an increase in membrane permeability to Na^+^ (**c**). In this way, the depolarization front moves. The subsequent increase in membrane permeability to K^+^ and reduction in permeability to Na^+^ returns the potential to negative values (**d**). In this way, a single depolarization front moves along the axon.

**Figure 7:**
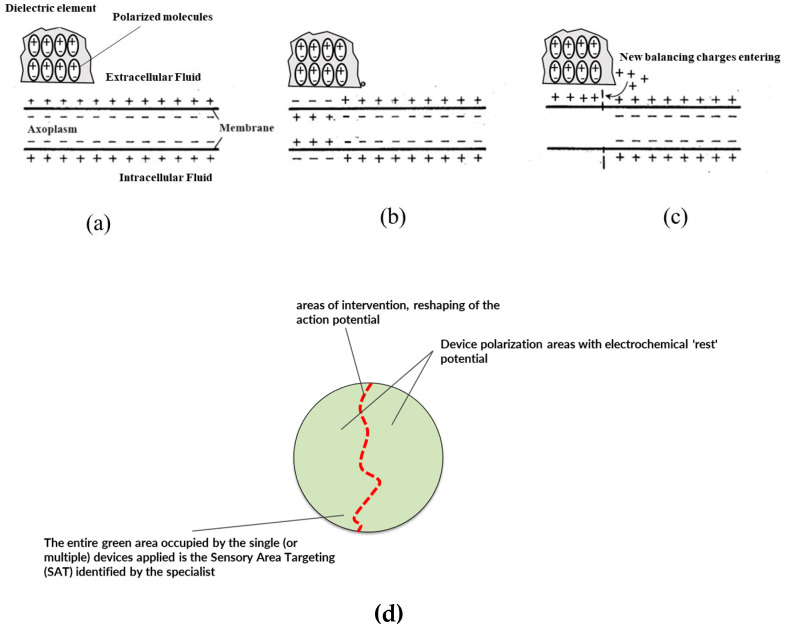
Schematic of the operating principle or mechanism of action of the capacitive device: (**a**) a cross-section diagram of an axon in conditions of resting membrane potential is visible; (**b**) the membrane, in a certain area (left side) is affected by depolarization, highlighted by the change in sign of the charges distributed along the portion of the membrane; (**c**) the dielectric internally orients its molecules due to electrostatic induction in coherence with the prevailing charge density, in this case in coherence with the positive charges of the membrane in rest conditions; (**d**) since the dielectric can orient the charges only in one direction, the dipoles created reflect the direction according to the prevailing charges, thus creating charge opposition—in the same way as a self-induced current generator—which acts with its own electromotive force (transport) on the charges present in the surrounding environment, re-establishing or in any case imparting a movement of the ionic charges of the boundary layer close to the membrane (repolarization effect of the membrane shown in Figure 4); (**d**) schematization of the principle of Area Sensory Target Approach: the specialist intervenes in an area where the neuron/nerve bundle affected by the transit of action potential trains to be remodulated is present. The neurons/nerves are represented by red lines. The application of the capacitive device in the area of interest where there is a signal disturbance of the trains of action potentials but also including a large “healthy” area where the distribution/density of ionic charges in the extracellular fluid is that of the system at rest, realizes the conditions referred to in (**b**,**c**) with a damping effect and reduction in potential peaks.

In summary, the interaction between an unpolarized dielectric and a positively charged electrolyte solution leads to the formation of an electric double layer, significantly altering the material’s internal electrical properties through dipole orientation and the external chemical properties of the electrolyte solution. The key therapeutic effect is based on the internal change in the electric properties of the material constituting the device. For a better understanding, we refer to Figure 6 and Figure 7. The two schematic views provide a comparison of what happens locally in afferent neuronal cells (i.e., epidermal terminals reporting information to the central nervous system) without (Figure 6) and with (Figure 7) the application of the claimed medical device.

Figure 6 illustrates the process of charge distribution and membrane potential changes in an axon during depolarization. Initially, positive charges accumulate on the outer surface of the axon membrane, maintaining the resting potential (Figure 6a). When a local depolarization is triggered and surpasses a certain threshold, the charge distribution in that specific area inverts (Figure 6b). This local inversion of charges causes an increase in membrane potential in adjacent areas, which also surpasses the threshold, leading to an increased permeability of the membrane to sodium ions (Na^+^) (Figure 6c). Consequently, a depolarization front moves along the axon. Following this, the membrane’s permeability to potassium ions (K^+^) increases while the permeability to Na^+^ decreases, returning the potential to negative values (Figure 6d). This sequence of events allows the depolarization front to propagate along the axon, enabling the transmission of nerve impulses.

Figure 7 depicts the situation of external stimulus and a reversal of the extracellular membrane polarity of the neural cell (i.e., peripheral nerve bundle). The reversal of membrane polarity is responsible for creating the above-threshold potential. However, the local presence in the target area—where the stimulus arises in the receptors—of the functional layer material with a predetermined dielectric constant polarizes passively and attracts more positive ionic charges from the extracellular fluid by electrostatic attraction. This phenomenon of ionic charge attraction in the extracellular fluid around the neural cell membrane is capable of restoring (or at least reducing) the local depolarization of the neural cell’s extracellular membrane. This phenomenon is due to the effective auto polarization of the material constituting the functional layer present in the invention’s medical device: the auto-polarization of the material of the dielectric layers claimed is responsible for the local response (see again Figure 7), causing the ionic attraction and the restoration or reduction in the action potential trains thus providing the technical effect.

One key technical distinguishing feature is to provide a simple passive function in which the dielectric material layers acts as an additional capacitive element that passively, and thus without a need of an external energy source, attracts additional positive charges from extracellular fluid of neural cell membrane thus restoring polarization or reducing neural cell membrane depolarization within the target areas.

The numerical model demonstrates that introducing an element with capacitive function, capable of interacting with the neuronal membrane (specifically with the extracellular fluid of the neuronal membrane) in critical intervention areas (the SAT points), can lead to signal remodulation and correction. This is evidenced by the modulation of the signal on a cartesian plane (millivolt versus milliseconds) shown in Figure 4, where the capacitor effectively remodulates the signals.

The technical effect on real cases, in comparison with numerical models, was therefore statistically investigated by the observational clinic study, structured to facilitate the comparison of the effect of an intervention on patients with PD against a control (placebo) group. The statistical tests applied to groups were matched for each data type (continuous vs. categorical). Differences in the mean age between the groups must be addressed in future studies, as it can influence the interpretation of the results and the overall conclusions drawn about the efficacy of the intervention. Except for age, the statistical analyses suggested no significant differences in most PD-related clinical parameters between the two groups.

Statistical analysis of the active (no placebo) group yielded significant insights into the therapeutic effects on various motor functions and risk factors associated with Parkinson disease.

Regarding the performance of the specific tasks in the BBS protocol, by results summarized in Table 3, we point out the following:

- *t*-statistic: This value measures how much the groups’ means differ in relation to the variability within the groups. A larger absolute t-value indicates a larger difference between the groups.
- *p*-Value: Shows the likelihood that the observed difference happened by chance. A value less than 0.05 indicates a significant difference.
- Significance, indicating whether the change is significant based on the *p*-value.

For the active group, the *t*-statistic of −3.72 and *p*-value of 0.0011 indicate a significant difference, meaning the observed changes are unlikely to have occurred by chance. For the non-active group, the *t*-statistic of 0.55 and *p*-value of 0.588 suggest that the differences observed are not statistically significant, indicating that they could be due to random variation.

Regarding the performance of the active group during the TUG tests, the Shapiro–Wilk test *p*-value show that most parameters had approximately normal distributions, with *p*-values above 0.05, except for P2 and P10.

Table 5 shows the statistical analysis result using the paired *t*-test that helps us understand how the treatment affected different aspects of patients’ mobility by comparing measurements taken before (T0) and after (T1) the treatment. Here below are listed the specific parameters that showed significant changes and what they mean for the dynamics of walking:

- *P1 (Stride Length):* Measures the length of each step. An increase in stride length indicates an improvement in the patient’s ability to walk with longer and smoother steps, which is often impaired in Parkinson’s patients.
- *P2 (Anteroposterior Acceleration—Lift Off):* Measures the forward/backward acceleration during the initial phase of standing up. A decrease in P2 suggests better control of lift-off movements, indicating greater efficiency and less effort required to initiate movement from a seated position.
- *P5 (Trunk Flexion–Extension during Lift-Off):* Measures the degree of trunk flexion and extension during lift-off. Improvements in P5 indicate better postural stability and control during lift-off, reducing the risk of falls.
- *P6 (Flexion Range):* Measures the range of trunk flexion movement during lift-off. An increase in flexion range indicates greater flexibility and movement capability, essential for stability and postural control.
- *P7 (Mid-turn Rotation):* Measures the duration of mid-turn rotation. A longer duration may reflect greater caution and control during rotational movements, which are often challenging for Parkinson’s patients.
- *P10 (Mid-turn Rotation Duration):* Measures the duration of mid-turn rotation. An increase in duration suggests that patients are more careful and balanced during turns, reducing the risk of falls.

For the non-active group of 25 patients who did not undergo therapeutic treatment, none of the parameters exhibited significant changes, as indicated by *p*-values above 0.05. The closest approach was P7 with a *p*-value of 0.166, suggesting no substantial effect attributable to the placebo.

Summarizing the comparative results between the active and non-active groups, different behaviors in terms of statistical significance and the magnitude of changes can be observed:

- Normality Assessment (Shapiro–Wilk Test): Most parameters in both groups followed a normal distribution, indicating the suitability of parametric tests for the analysis.
- Paired *t*-Test Results: The active group showed significant changes in several parameters post-treatment, indicating the effectiveness of the therapeutic intervention. In contrast, the non-active group showed no significant changes, suggesting the placebo had no measurable effect.
- The statistical analysis of the placebo group shows the absence of a therapeutic effect from non-active treatment, contrasting with the active group, where certain parameters exhibited significant improvements. This highlights the effectiveness of the therapeutic intervention in improving specific aspects of motor function in patients with Parkinson’s disease. In particular, there is a significant reduction in the risk of falling and improvements in both anteroposterior accelerations and flexion–extension of the shaft, which are crucial for maintaining stability and mobility in patients with Parkinson’s disease.

Ultimately, the application mapping for device placement is key to producing effects on the mobility-related parameters.

As major outcome from patients’ perspectives, one major advantage of the medical device employed in this study is that it does not require to be powered by any internal or external energy source and does not release any substance into the body nor extract drugs, bodily fluids, or any other substances from the body.

## 5. Conclusions

This study aimed to evaluate the efficacy level of the capacitive autonomous wearable device (i.e., passive sensor, not powered or sourced) in modulating neuromuscular activity in Parkinson’s patients. The device was applied at five critical intervention points, specifically selected by therapists to target key muscles involved in posture and movement. The main advantages over other non-invasive methodologies are:

▪ Localized intervention without systemic side effects, improved patient compliance due to the non-invasive nature, and continuous therapeutic effects.
▪ Compared with other non-invasive, non-pharmaceutical treatments such as magnetic cyclotron resonance, laser treatments, and anodic/cathodic current applications (all mentioned in the introduction), this wearable solution, characterized by the passive action of the device (no power source needed), allows patients to continue therapeutic treatment autonomously after the initial study and application by a therapist.
▪ This targeted modulation helps to progressively correct the aberrant action potential trains responsible for impaired motor function in Parkinson’s patients over prolonged application times of up to 8 h per day.
▪ The wearable nature of the device provides continuous therapeutic effects without the need for repeated clinical visits, thus improving patient compliance and overall quality of life.

The results from our observational study demonstrate significant improvements in several key parameters of motor function, including stride length, anteroposterior acceleration, trunk flexion–extension, and mid-turn rotation duration. These improvements were statistically significant in the active treatment group compared to the placebo group, indicating the device’s efficacy in enhancing mobility and reducing the risk of falls.

The wearable passive sensor used in this pilot study represents a non-invasive and non-pharmacological approach for managing Parkinson’s disease, offering a valid alternative to existing therapies. The passive nature of the device ensures minimal interference with the patient’s daily activities, enhancing compliance and quality of life.

The future purpose of our capacitive wearable device is to serve as a complementary therapy for Parkinson’s disease management, not as a substitute for pharmacological treatment. We aim to integrate this device into standard care protocols to enhance motor function and reduce symptoms such as rigidity and bradykinesia. Future developments will focus on optimizing the device for broader clinical use, including refining its design for better comfort and ease of use, and conducting larger-scale clinical trials to further validate its efficacy. Ultimately, we envision this device becoming a standard tool in the therapeutic arsenal against Parkinson’s disease, potentially extending its application to other neuromuscular disorders.

While the initial results are promising, it is important to acknowledge the limitations of this preliminary study, including the sample size. Further large-scale studies are needed to confirm these findings and to solidify the device’s role in the therapeutic landscape for neurodegenerative diseases.

## 6. Patents

The three authors of this article are listed as inventors on the Italian patent granted for the capacitive medical devices discussed in this manuscript. Additionally, two applications for patents in the European Union and the United States are currently pending. These patents cover the technologies and methodologies used, which are discussed in this study.

## Figures and Tables

**Table 1:** Comparative analysis of clinical characteristics between non-placebo patients, namely the active group, and placebo patients, namely the non-active group in the Parkinson’s Disease Pilot Study. For BMI, disease duration, MMSE, daily levodopa dose (LED) and age characteristics of groups, the Mann–Whitney U statistical test has been used; for the H&Y scale characteristic, the chi-square test has been selected.

	Active Group						Placebo Group						
Characteristic	(*n*: 25)						(*n*: 25)						
Gender	12 M; 13 F						14 M; 11 F						
	Mean	St.Dev	Min	Max	25%	75%	Mean	St.Dev	Min	Max	25%	75%	*p*-Value
BMI	23.78	3.99	17.83	33.33	21.80	25.71	23.54	4.75	14.33	35.22	20.40	25.59	0.685
Disease duration	14.18	6.31	5.00	27.00	9.00	19.89	14.50	4.72	7.00	25.00	11.25	17.75	0.624
MMSE	25.86	1.25	24.00	27.50	25.00	27.00	25.45	1.06	23.50	27.40	24.60	26.10	0.282
Daily levodopa dose (LED)	631.6	160.4	380.0	890.0	480.0	740.0	613.1	140.4	390.0	890.0	497.5	745.0	0.727
Age	77.56	5.85	64.00	91.00	74.00	81.00	67.15	7.00	56.00	83.00	63.00	71.75	4.112 × 10^−6^
H&Y scale	2.98	0.73	2.00	4.00	2.50	4.00	3.15	0.61	2.50	4.00	2.50	4.00	0.607

**Table 2:** Summary of parameters measured in clinical trials for Parkinson’s disease patients using instrumented Time Up and Go Tests (equipped with an accelerometer). This table presents the parameters assessed both at baseline (T0, without stabilizer) and after the application of stabilizer (T1). Measurements included duration and acceleration during specific movements, risk of fall, trunk flexion–extension during both lift-off and sitting-down phases, and rotations, highlighting the changes between the T0 and T1 phases to understand the impact of the stabilizer.

Parameter Description	Unit of Measure	Parameter Code
Duration of the Test	s	P1
Anteroposterior Acceleration—Lift Off	m/s	P2
Anteroposterior Acceleration—Sitting Down	m/s	P3
Fall Risk	-	P4
Trunk Flexion–Extension during Lift Off—Peak Flexion	degrees	P5
Trunk Flexion–Extension during Lift Off—Flexion Range	degrees	P6
Trunk Flexion–Extension during Sitting Down—Peak Flexion	degrees	P7
Trunk Flexion–Extension during Sitting Down—Peak Extension	degrees	P8
Flexion Range	degrees	P9
Mid-Turn Rotation	s	P10
Final Rotation	s	P11

**Table 3:** Results of a paired *t*-test analyzing pre-treatment and post-treatment BBS scores within the active and inactive groups.

Group	Test Used	*t*-statistic	*p*-Value	Significance
Active Group	Paired *t*-test	−3.72	0.0011	Statistically significant (*p* < 0.05)
Non-Active Group	Paired *t*-test	0.55	0.588	Not statistically significant (*p* > 0.05)

**Table 4:** The Shapiro–Wilk test was used to verify the normality of the measured parameters for the active group.

Geit Analysis Parameter (TUAG Protocol)	Shapiro–Wilk Statistic	Shapiro–Wilk *p*-Value
P1	0.931	0.090
P2	0.888	0.010
P3	0.965	0.529
P5	0.915	0.039
P6	0.960	0.407
P7	0.939	0.144
P8	0.945	0.192
P9	0.950	0.255
P10	0.899	0.017
P11	0.923	0.059

**Table 5:** The results of the paired *t*-test highlighted significant variations between the pre- and post-treatment measurements for each parameter (active group).

Geit Analysis Parameter (TUAG Protocol)	Test *t* Statistic	Test *t* *p*-Value
P1	2.367	0.026
P2	−3.326	0.003
P3	−1.934	0.065
P5	−2.644	0.014
P6	−2.439	0.023
P7	−2.274	0.032
P8	−1.111	0.278
P9	−2.229	0.035
P10	2.853	0.009
P11	0.725	0.475

**Table 6:** The Shapiro–Wilk test was used to verify the normality of the measured parameters in the non-active group.

Geit Analysis Parameter (TUAG Protocol)	Shapiro–Wilk Statistical	Shapiro–Wilk *p*-Value
P1	0.970	0.616
P2	0.961	0.408
P3	0.974	0.729
P5	0.950	0.227
P6	0.929	0.073
P7	0.899	0.015
P8	0.982	0.909
P9	0.954	0.287
P10	0.963	0.463
P11	0.985	0.964

**Table 7:** The results of the paired *t*-test highlighted significant variations between pre- and post-treatment measurements for each parameter (inactive group).

Geit Analysis Parameter (TUAG Protocol)	Test *t* Statistic	Test *t* *p*-Value
P1	0.397	0.695
P2	−0.118	0.907
P3	1.227	0.231
P5	−0.337	0.739
P6	−1.078	0.291
P7	1.425	0.166
P8	0.893	0.380
P9	1.004	0.325
P10	−1.118	0.274
P11	−1.329	0.196

## Data Availability

The raw data supporting the conclusions of this article will be made available by the authors on request.
